# Effects of a cancer prevention education program on elementary school students’ knowledge, attitude, self-efficacy, and intentions in South Korea

**DOI:** 10.4178/epih.e2019027

**Published:** 2019-06-16

**Authors:** Su Yeon Kye, Soon-Yong Hwang, Kyung Hee Oh, Jae Kwan Jun

**Affiliations:** 1 Cancer Information and Education Branch, National Cancer Control Institute, National Cancer Center, Goyang, Korea; 2 Biostatistical Consulting Lab, Medical Science Research Center, Korea University College of Medicine, Seoul, Korea

**Keywords:** Cancer prevention education, Elementary school, Program evaluation, Quasi-experimental design

## Abstract

**OBJECTIVES:**

Most children and adolescents have low levels of cancer knowledge and awareness, and infrequently engage in preventive behaviors. This study examined the effects of a short classroom-based intervention for cancer prevention on knowledge, attitude toward cancer preventability, self-efficacy, and behavioral intentions of fifth-grade elementary school students.

**METHODS:**

The study was based on a pre-post-follow-up, 2-group, quasi-experimental design. Participants in the intervention group attended two 40-minute sessions on cancer prevention education and watched a music video about cancer prevention, while participants in the control group were only exposed to the music video. Self-reported knowledge, attitude toward cancer preventability, self-efficacy, and behavioral intentions were assessed 1 week pre-intervention and post-intervention, as well as 3 months post-intervention.

**RESULTS:**

The 3-month post-intervention results revealed partial effects, indicating that the education intervention improved knowledge and attitudes toward cancer preventability; however, no effects were observed on self-efficacy and behavioral intentions 3 months after the intervention.

**CONCLUSIONS:**

Long-term regular booster sessions are required to improve not only social-cognitive factors, but also behavioral intentions, which could result in behavior changes promoting cancer prevention.

## INTRODUCTION

Although cancer is a major cause of morbidity and mortality worldwide, the World Health Organization (WHO) estimates that one-third of the overall incidence of cancer could be prevented if certain individual lifestyle choices, such as smoking, limited physical activity, unbalanced diet, and alcohol consumption, were improved [1].

Childhood and adolescence are marked by an increasing involvement in health-risk behaviors, and if established, these behaviors can adversely influence health in the long term [2]. According to the Korean Youth Risk Behavior Web-based Survey, nearly 15% of high school students smoke cigarettes on a regular basis and an even higher proportion of students 25% consume alcohol regularly. Furthermore, more than 80% of high school students do not exercise regularly and 15% are obese [3].

Several studies have suggested that most children and adolescents have low levels of cancer knowledge and awareness, and infrequently engage in preventive behaviors [4]. Because the alteration of routine risk behaviors becomes more difficult as people grow older, it is necessary to deliver interventions to promote healthy lifestyle choices as part of a child’s development [5]. Children and adolescents can be easily accessed through schools, and schools are therefore a useful arena for improving their cognitions and behaviors regarding cancer prevention. The WHO identified schools as playing a central role in instilling health promotion and education activities [6]. However, cancer education programs in schools generally either focus on only a single behavioral risk factor or are mainly designed to improve cancer awareness, although they include the comprehensive topic of risk factors for cancer [5,7-9]. In South Korea (hereafter Korea), education, even at the elementary school level, is centered on college entrance examinations, with classes focusing on the subjects covered in entrance examinations. Thus, no specific education program focuses on cancer prevention, and although some developed countries include health education in their national health plans, it is difficult to allocate many school hours for health education [10]. Therefore, we developed a short-term comprehensive cancer prevention education program, called “Health UP & Cancer DOWN,” that targeted multiple behavioral risk factors for cancer among fifthgrade elementary school students. The overall frame of this program was based on social cognitive theory, which emphasizes increasing behavioral capability, outcome expectations, and self-efficacy. According to social cognitive theory, people learn by observing others, learning occurs through the reciprocal triadic relationship of environment, individual, and behavior, and learned behavior can become central to one’s personality [11]. Skills to improve students’ knowledge, attitude, self-efficacy, and intentions were derived from the behavior change techniques (BCT) taxonomy, which lists potentially effective techniques for inclusion in behavior change interventions across behavioral domains [12]. The objectives of this program were to promote the knowledge of cancerrelated risk factors and positive attitudes toward cancer preventability and to increase self-efficacy and intentions to engage in cancer-preventive behaviors.

This study examined the effects of a short, classroom-based cancer preventive intervention on knowledge, attitude toward cancer preventability, self-efficacy, and behavioral intention of fifth-grade elementary school students.

## METHODS

### Design

This study was based on a pre-post-follow-up, 2-group, quasiexperimental design to examine the effects of cancer prevention education on knowledge, attitude toward cancer preventability, self-efficacy, and behavioral intentions of cancer-preventive behaviors among elementary school students.

### Ethics statement

All procedures performed in studies involving human participants were in accordance with ethical standards of the institutional and/or research committee and with the 1964 Helsinki declaration and its later amendments or comparable ethical standards. The study protocol was granted by the Institutional Review Board of the National Cancer Center (IRB no. NCC2017-0005). Informed consent: Informed consent was obtained from all individual participants included in the study.

### Participants

**Figure 1.**
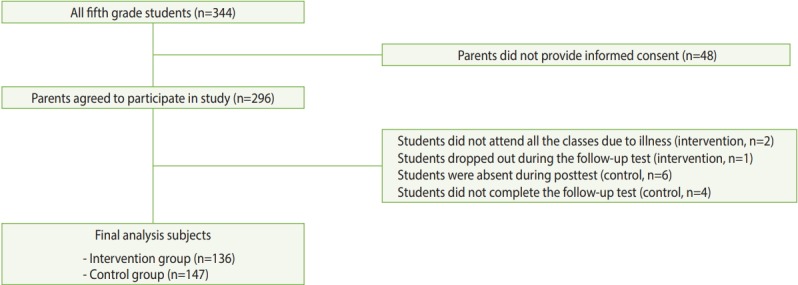
Flowchart of the participant selection.

The participants comprised fifth-grade elementary schoolchildren at 4 schools located in the city of Goyang, outside Seoul, Korea. Researchers asked the Goyang Office of Education to recommend eligible schools for the program. The 4 selected schools had similar gender proportions and socioeconomic backgrounds. All fifth-grade students at the 4 selected schools were studied. The participants were randomly assigned to either the intervention group or the control group at the school level to avoid contamination, using a random number table. The schools, located in different districts, were approximately 15 minutes apart by car. Consequently, students from each school were unlikely to interact with each other. The educational program was delivered to the participants in the intervention group. There were no exclusion criteria for the students. Within the participating schools, consent forms regarding pre-intervention, post-intervention, and follow-up assessments were distributed to the parents of all 344 fifth-grade students. A total of 296 (86.0%) parents provided informed consent for their children to be included in the study. In the intervention group, 2 students did not attend all the classes due to illness and 1 student dropped out during the follow-up test; thus, a total of 136 (97.8%) students were eligible for the final analysis. In the control group, 147 (93.6%) questionnaires were included in the final analysis, excluding 6 students who were absent during the post-test and 4 students who did not complete the follow-up test (Figure 1).

### Procedure

The participants in the intervention group received 2 sessions of cancer prevention education and watched a music video about cancer prevention, while those in the control group were exposed twice to the music video only. The educational intervention was administered weekly for 2 weeks by a school nurse at each school during regular classes (40 minutes in Korean elementary schools) in groups of up to 25 participants in November 2016. The music video was shown during the second session for the intervention group and during the same week for the control group by a school nurse. The 4 school nurses had more than 10 years of experience in both health care and health education at the elementary school level and had participated in the development of the intervention program. For the intervention group, the pre-test (T_0_) was conducted 1 week before the intervention and the post-test (T_1_) was completed 1 week after the intervention; the follow-up test (T_2_) was administered 3 months after the intervention. All tests were administered at the same times for the control group. Before the assessments, the participants were informed about the aims of the study by the form teacher and anonymity was assured. After the follow-up test, cancer prevention education was provided to the control group participants.

### Intervention

The intervention was an educational program entitled “Health UP & Cancer DOWN.” The content of the cancer prevention education program was designed by the researchers and 4 school nurses who specialized in health education and school nursing. The curriculum covered cancer and lifestyle factors associated with cancer prevention. Its 2 sessions were titled “What is cancer?” and “Is cancer preventable?” The first session focused on the definition, types, causes, symptoms, and treatment of cancer and the second session addressed lifestyle choices that prevent cancer, such as non-smoking, non-drinking, consuming fruits and vegetables, avoiding processed meat or charred foods, engaging in physical activity, and maintaining a healthy weight. The curriculum was based on social cognitive theory, with the goal of improving behavioral capability, attitudes toward cancer preventability, outcome expectations, and self-efficacy. As an educational strategy, we used the BCT taxonomy [12]. The first session targeted knowledge, attitudes toward cancer preventability, and outcome expectations, using the techniques of providing information on the behavior–health link and consequences and motivational interviewing. Through quizzes and presentations, the participants learned the characteristics of cancer, and in a roleplaying activity, they imagined that they were doctors and explained the importance of cancer prevention to their partners. The second session focused on self-efficacy and intention improvement and applied the techniques of barrier identification, prompting intention formation, and agreement on a behavioral contract. Through a group discussion, the participants identified barriers to cancerpreventive behaviors and discussed possible solutions. In addition, the participants developed guidelines that they could comply with for cancer prevention and wrote messages of support for one another (Table 1). Lesson plans, teaching materials, and a handbook were developed and distributed to the teachers and students of the intervention group, respectively. An 80-second music video was developed by the national educational broadcasting system and covered the health behaviors for cancer prevention through a song with a catchy hook.

### Measures

The survey instrument was a self-administered anonymous questionnaire with 21 items, assessing demographic variables, knowledge, attitudes toward cancer preventability, self-efficacy, and behavioral intentions for cancer prevention. To measure levels of knowledge, self-efficacy, and intention, the research team selected 6 items based on the Cancer Prevention Codes developed by the Korea National Cancer Center: non-smoking, non-drinking, consuming fruits and vegetables, avoiding processed meat and charred foods, engaging in physical activity, and maintaining a healthy weight. To measure participants’ knowledge, they were asked to rate the level of helpfulness of each behavior for cancer prevention on a 5-point Likert scale. Self-efficacy was measured by rating participants’ level of confidence in their own ability to engage in cancer prevention behaviors on a 5-point Likert scale. Intentions were assessed by having participants rate their level of willingness to comply with each behavior on a 5-point Likert scale. The respective Cronbach alpha coefficients for knowledge, self-efficacy, and behavioral intentions were 0.728, 0.736, and 0.782 at the pre-test, 0.852, 0.795, and 0.815 at the post-test, and 0.850, 0.809, and 0.846 at the follow-up test. Attitudes toward cancer preventability were assessed by rating the statement “cancer is preventable” on a 5-point Likert scale.

### Analyses

The data were analyzed using SPSS version 19.0 (IBM Corp., Armonk, NY, USA). The Pearson chi-square test and the independent t-test were used to examine differences between the intervention and control groups at baseline (T_0_). For intervention effects, repeated-measures analysis of variance (ANOVA) was used to compare the outcome scores of knowledge, attitudes toward cancer preventability, self-efficacy, and behavioral intentions between the intervention and control groups at 1 week pre-intervention (T_0_), 1 week post-intervention (T_1_), and 3 months postintervention (T_2_).

## RESULTS

**Figure 2.**
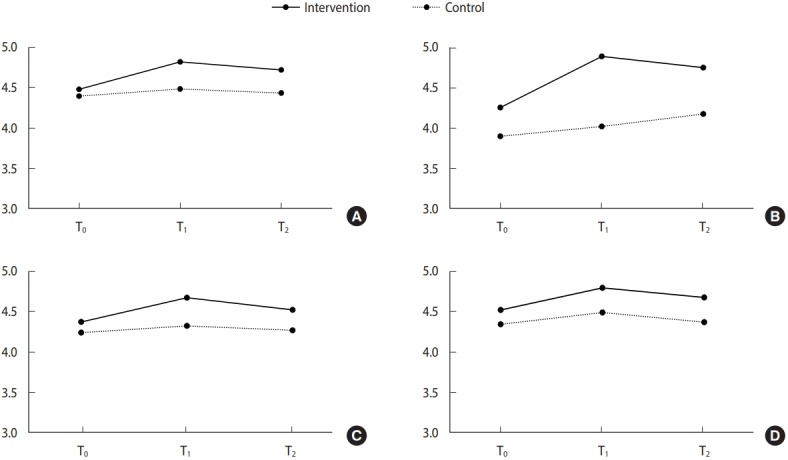
Comparison of the pre-, post-, and follow up-test mean for (A) knowledge, (B) attitude toward cancer preventability, (C) selfefficacy, and (D) behavioral intention. T_0_, pre-intervention; T_1_, post-intervention; T_2_, 3-month post-intervention.

A total of 283 participants completed the questionnaires at the 3 time points. The average age of the participants was 11 years and 46.3% were female. At baseline (T_0_), no significant differences were found for sex, knowledge, and self-efficacy, whereas the scores for attitude toward cancer preventability and behavioral intentions were significantly higher in the intervention group than in the control group (M_attitude to cancer preventability_= 4.26, 3.87; M_behavioral intentions_= 4.51, 4.33; p_attitude to cancer preventability_= 0.001; p_behavioral intentions_= 0.010). Repeated-measures ANOVA indicated statistically significant differences between the 2 groups (p< 0.001) and across the 3 time points (p < 0.001). An interaction effect was observed between group and time for knowledge (p< 0.001), attitude toward cancer preventability (p< 0.001), and self-efficacy (p= 0.003). For both knowledge and attitude toward cancer preventability, significant interaction effects were observed between the groups and time from T_0_ to T_1_ (p< 0.001, p< 0.001, respectively) and from T_0_ to T_2_ (p= 0.005, p= 0.040, respectively), thereby highlighting the benefit of cancer prevention education over time. However, regarding self-efficacy and behavioral intentions, no interaction effects were observed from T_0_ to T_2_ (p= 0.086, p= 0.066, respectively), although significant interaction effects were observed from T_0_ to T_1_ (p<0.001, p= 0.025, respectively) (Figure 2 and Table 2).

## DISCUSSION

This study conducted a quasi-experimental evaluation of cancer prevention education among elementary school students. The results revealed partial effects, indicating that the education improved knowledge and attitude toward cancer preventability 3 months after the intervention; however, no effects were observed on self-efficacy and behavioral intention 3 months after the intervention.

One week post-intervention, the educational intervention significantly increased all outcome variables. Among these measures, the improvements in knowledge and attitude toward cancer preventability were maintained 3 months post-intervention. Social cognitive factors, such as belief, outcome expectations, self-efficacy, intention, and practice, require more complicated processes and longer periods for improvement than knowledge change, as these factors are more difficult to influence [7,8]. Previous studies reporting knowledge improvements have conducted short-term intervention programs once or thrice a week for 30 minutes to 1 hour [4,7-9,13]. In contrast, interventions with a significant effect on behavioral intentions and practice required a longer time. A full-time 5-day intervention was effective in increasing intentions to engage in health-promoting behavior, as well as increasing knowledge about cancer and risk factors for cancer, and a 7-month nutritional intervention significantly improved the dietary self-efficacy of children [14,15]. A healthy life practice education program with 10 sessions targeting elementary school students in Korea showed significant differences in health behaviors for disease prevention and safety [16]. The lack of significant differences in self-efficacy and behavioral intention between the intervention and control groups in this study could, to a large extent, be attributed to the duration of the educational intervention, which may have been too short for a significant change. In other words, although knowledge may impact decision-making regarding cancer prevention and reduce some behavior-related cancers in adulthood, long-term regular booster sessions are required to improve social cognitive factors that may have a greater influence on changes in cancer prevention behavior.

The study has several limitations. First, the generalizability of these findings is limited because the study included schools from a single small area in Korea. Moreover, no randomization procedures were used for the selection of both target schools and individual participants. Therefore, the influence of the confounding factors that were not assessed cannot be ruled out. Moreover, selfreports are prone to certain types of biases, such as social desirability. Finally, a 3-month follow-up period was chosen primarily to complete the study within the academic year. Thus, the findings of this study do not indicate how long the improvements in knowledge and attitude toward cancer preventability resulting from this intervention would be maintained over a longer period.

To our knowledge, this is the first study in Korea to evaluate early adolescents’ knowledge and social cognitive variables using an intervention for cancer prevention. It is novel because it evaluated education on several known cancer risk factors concurrently. Previous studies have focused on preventive interventions for single risk factors [5,8,15,17]. By applying standardized behavior change techniques within the framework of the health behavior change model, it could be possible to develop intervention programs more systematically; namely, this program can be replicated in other populations with different ages and cultures, and any differences in the results could be attributed to the differences in the population, rather than the method. Another advantage of this brief educational intervention is that it developed lesson plans and teaching materials for teachers and a handbook for students, as well as a curriculum that could be easily implemented in a school environment or community health service settings.

This study, therefore, provides support for the further development and testing of this brief intervention, which led to significant increases in social cognitive factors at 1 week post-intervention, not all of which were maintained 3 months post-intervention. Moreover, additional intervention studies involving a greater number of students and schools with randomized sampling are needed to address the limitations introduced by the sampling approach; this would also enable a subgroup analysis by socio-demographic factors and the degree to which participants engage in health behaviors. Furthermore, since the ultimate goal of health education, beyond the alteration of social cognitive factors, is behavior change, further studies evaluating cancer-preventive behaviors over the long term are required.

Children can be easily accessed through schools, making schools a useful target for interventions designed to improve cognitions related to cancer prevention. Long-term regular booster sessions will be required to improve both social cognitive factors and behavioral intentions, which could lead to behavior changes for cancer prevention. In addition, in designing interventions for cancer prevention, evidence-based behavior change techniques can be useful to reduce the time and resources invested by developers and educators. Despite the failure of this intervention to lead to a lasting effect on social cognitive factors and intentions, the results suggest that short-term cancer prevention education using proven techniques is effective in improving cancer knowledge and attitudes toward cancer preventability, and that such interventions should be encouraged.

## Figures and Tables

**Table 1.** Content of the cancer prevention education program

Session	Topic	Content	Techniques	Methods
1	What is cancer?	Definition	Providing information about behavior-health risk and consequences	Quiz
		Types		Presentation
		Causes	Motivational interviewing	Role-play
		Symptom		
		Treatment		
		Importance of cancer prevention		
2	Is cancer preventable?	Non-smoking	Barrier identification	Group discussion
		Non-drinking	Promoting intention formation	Action plan
		Consumption of vegetables and fruits	Agreement on behavioral contact	Music video
		Avoiding processed meat and charred foods		
		Engaging in physical activity		
		Maintaining a healthy weight		

**Table 2.** Effects of cancer prevention education in elementary school students

Variable	Group	T_0_	T_1_	T_2_	Effects	F-value	p-value
Knowledge	Intervention	4.47±0.45	4.82±0.32	4.72±0.38	Group	22.30	<0.001
	Control	4.39±0.54	4.48±0.61	4.43±0.65	Time	24.31	<0.001
					Group×time	9.14	<0.001
					T_0_-T_1_	18.84	<0.001
					T_0_-T_2_	8.04	0.005
					T_1_-T_2_	0.77	0.380
Attitude toward cancer pre-ventability	Intervention	4.26±0.78	4.90±0.32	4.75±0.49	Group	63.74	<0.001
	Control	3.87±1.09	4.03±1.01	4.16±0.83	Time	38.00	<0.001
					Group×time	12.93	<0.001
					T_0_-T_1_	20.49	<0.001
					T_0_-T_2_	4.26	0.040
					T_1_-T_2_	13.01	<0.001
Self-efficacy	Intervention	4.36±0.55	4.67±0.35	4.52±0.47	Group	18.99	<0.001
	Control	4.23±0.63	4.32±0.62	4.26±0.67	Time	18.38	<0.001
					Group×time	5.90	0.003
					T_0_-T_1_	14.07	<0.001
					T_0_-T_2_	2.96	0.086
					T_1_-T_2_	2.47	0.117
Behavioral intentions	Intervention	4.51±0.52	4.78±0.32	4.66±0.40	Group	25.20	<0.001
	Control	4.33±0.60	4.48±0.58	4.36±0.64	Time	22.46	<0.001
					Group×time	2.79	0.065
					T_0_-T_1_	5.06	0.025
					T_0_-T_2_	3.39	0.066
					T_1_-T_2_	0.01	0.998

Values are presented as mean±standard deviation. T_0_, pre-intervention; T_1_, post-intervention; T_2_, 3-month post-intervention.
